# Search engine optimisation (SEO) strategy as determinants to enhance the online brand positioning

**DOI:** 10.12688/f1000research.73382.1

**Published:** 2022-06-28

**Authors:** Umar Faruq Ahmad, Junainah Mahdee, Normazalila Abu Bakar

**Affiliations:** 1 Corporate Communication Division, Ministry of Home Affairs, Federal Government Administrative Centre, Putrajaya, Wilayah Persekutuan, 62546, Malaysia; 2 Faculty of Management, Multimedia University, Cyberjaya, Selangor, 63100, Malaysia; 3 MM Smart Earth Resources, Seri Kembangan, Selangor, 43300, Malaysia

**Keywords:** SEO, online, brand, positioning, determinants, marketing, strategy

## Abstract

**Background**– Marketers face evolution of online brand positioning marketing strategy due to changes of search engine algorithm that affects the reaching out of brands to potential internet users. Brand owners realise that to be relevant in modern market, they need to transition and focus more into online market. However, many brand owners have ignored the power of search engine optimisation (SEO) strategy for attracting the online market, which is highly competitive and faces rapid changes.  A brand can be considered as old fashioned if it does not utilise the SEO as their marketing strategy, in penetrating the online marketplace. Various studies have analysed factors that can enhance the persistency of using the SEO strategy, however gaps remain regarding the relationship of this strategy with the online brand positioning. The main aim of this study was to identify the persistency of using the SEO strategy including the niche point of differentiation, valuable content, targeted keyword and scalable link building, as the determinants that enhance the success of online brand positioning.

**Methods**– This study applies quantitative design using online survey to gather information from the online business entrepreneurs. The survey questionnaire was arranged to focus on the use of SEO as a new way to strategies online business.

**Results**– Based on the results of this study, most online entrepreneurs have somewhat realised the effects of using the SEO strategy to enhance effectiveness of online brand positioning.

**Conclusion**– This research provides insights on the importance of SEO strategy in online business positioning. It is hoped that the online entrepreneurs will take the SEO strategy into consideration in the positioning of their brand in the marketplace.

## Introduction

Search Engine Optimisation (SEO) incorporates technicality and creativity elements that enable online entrepreneurs to increase users’ traffic, thus upgrade its rankings in search engines utilisation. ^1^ Online entrepreneurs are knowledgeable with up-to-date technologies in marketing and selling products via online platforms. Online entrepreneurs must adapt with the online evolution especially search engines because any changes of search engine algorithm will affect brands’ reach out to potential buyers. To stay relevant in the modern market, brand owners must be ready to migrate from conventional to online business, with more focus towards online brand strategy. ^1^

The aim for any marketing campaign is finding the right online brand marketing strategy, e.g., defining the right marketing message to influence consumers on their brands. The technical and creativity aspects of digital marketing will enable online entrepreneurs to improve their brand position in online search engine platforms. ^2^ Online entrepreneurs must also have the ability to quickly adapt with SEO strategies to position their brands effectively.

Due to the rapid changes of technology and tough competition from rivals, many brand owners have opted to ignore the power of SEO in their online brand positioning strategy. This has caused a big loss to the brand owners for not taking advantage of the “limitless world” of internet for their marketing strategies. The SEO saves a lot of money to reach the highest numbers of customers compared to other marketing methods that has limited reach.

Various studies related to the factors that enhance the persistency of using a SEO strategy have been performed, however the use of this strategy as determinants to enhance online brand positioning has not been investigated. As such this study analysed the online brand positioning for entrepreneurs using the SEO strategy for longevity in the modern market. Specifically, the objective was to investigate the influence of being persistent by using niche point of differentiation, valuable content, targeted keyword, and scalable link building, in SEO strategy among the online entrepreneurs.

## Literature review

Online business has been referred to as “ *as the process of buying, selling, or exchanging products, services, and or information via computer network, including the internet*”. ^3^ Online business consists of business transactions via online whereby sellers can advertise, promote, and sell their items via internet. In comparison to the traditional brick-and-mortar, online business provides 24-hours services that are operating throughout the year. Online platforms enhance business market position and its presence to the target audience. ^4^ The most crucial part for an online business success is the effective information technology and communication system in the World Wide Web for serving customers. ^5^^,^^6^

Online business attracts new markets, thus enhancing the productivity and performance of firms. ^7^^–^^11^ This type of business was not only adopted by huge firms but also small businesses globally. ^12^ Conventional off-line businesses that are perceived as a growing new market are also attracted to move to online platforms. ^13^ Since online business is relatively new, entrepreneurs must consider several barriers such as security and privacy, dependability on technology, maintenance cost, hectic online price and competition; especially for small sizes online entrepreneurs. ^14^^–^^17^ Despite all obstacles and challenges facing online business, there are many innovative opportunities that online business entrepreneurs can venture into, to ensure their success ^18^; inclusive of trust, cyberlaw and cybersecurity, mobile commerce, e-warranty, effective services deliveries, prompt and easy payment schemes and many others which are better than conventional business transactions. ^19^

Additionally, online business can take advantage from the rapid increase of Internet user base, positive mindset, especially among youngsters towards easier online shopping regardless of time and location. ^20^

The global increase of Internet penetration and enhancement of legal aspects of online business can grow online businesses in any country. ^21^ Search engines support online business strategies; however they are not designed for online business and consumers to search anything by browsing just one web page. There are many search engines available with main key players dominated by well-known names such as Google, Yahoo, and Bing. The search engines have been used by consumers to find anything online, therefore they have become a new platform for businesses opportunities. ^22^ For a brand to be perceived as popular, the SEO strategy targets for the brand name to be listed on the first page when a keyword is entered in the search engine. Normally, customers will not scroll down to the bottom of the first page, or they will not visit too many pages due to time constraint. ^23^ Online entrepreneurs must acknowledge the importance of dependency on the SEO strategy to launch brand campaigns, so that target audience can choose their brand despite the competition. The review of the relevant literature has indicated that there are four major components of the SEO strategy: niche point of differentiation, valuable content, targeted keyword, and scalable link building to enhance online brand positioning. ^24^

### Niche point of differentiation

Niche marketing with the use of SEO strategy means the utilisation of the online advertising strategy that focuses on specific group of customers as the niche market; to enable the full benefits to the target markets at the lowest costs and fastest coverage as possible. ^25^ The differentiation of niche could give advantage to a brand that has particular strategies in online marketing and branding. Not only that, the niche can also be determined by where the brand is positioned in the search engine competition. ^25^

### Valuable content

A fresh page with quality content is more valuable than old contents. Many scholars agreed that ‘Content is King’, whereby any good content will link with other content automatically. High rated content results in high quality search engine optimisation. ^26^ Relative positioning of the content and the quality of the content are the main elements of the valuable content factors. In addition, the frequency of the content will give ‘bonus mark’ to the search engine. ^27^

### Targeted keywords

Specific placement of targeted keywords for the brand will enhance the effectiveness of the SEO in online brand positioning. ^28^ An organic branded keywords traffic naturally comes from the search engine. Therefore, discovering certain related keywords to the brand that are always being searched is crucial in online branding. Another plus point is that integrating keywords can create a competitive advantage to the online brand. ^29^ Keywords can bring direct competition for the higher-ranked slots. Site visibility in Google search can be significantly improved by using targeted keywords. ^30^

### Scalable link building

Scalable link building is a process whereby consumers start with a generic keyword when searching for any products, and later become more specific to certain brand’s uniqueness. ^31^ Navigational link with other online retailers will associate the brand positioning in the online market. Increasing number of links to the online brand placement and link architecture can give some additional information to the search engine web crawlers. Anchor links are recognised as important factors in Google’s algorithm. ^32^

### Online brand positioning

Online brand positioning via SEO is affected by the numbers of key contextual factors. ^33^ Online marketers recognises that ranking of the brand is effectively enhanced by brand positioning in the online market. ^34^ By associating with the big brands or market leaders of the industry in the same platform using a search engine will also increase the brand value for the consumers. ^35^

**Figure 1.**
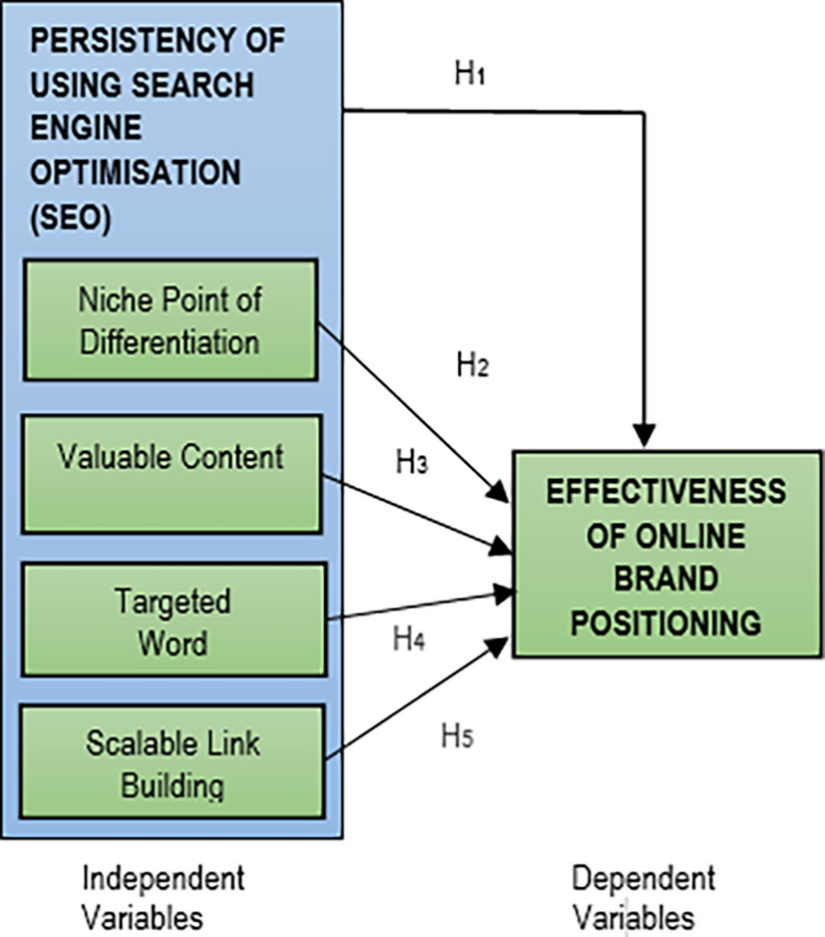
Research theoretical framework.

Based on the deliberations of the SEO components and the online brand positioning, the research theoretical framework is illustrated in Figure 1.

Five hypotheses were developed based on this research theoretical framework:

H _1_: There is a significant positive relationship between the persistency of using SEO strategy with the success of online brand positioning.

H _2_: There is a significant positive relationship between the persistency of using niche point of differentiation in SEO strategy with the success of online brand positioning.

H _3_: There is a significant positive relationship between the persistency of using valuable content in SEO strategy with the success of online brand positioning.

H _4_: There is a significant positive relationship between the persistency of using targeted keyword in SEO strategy with the success of online brand positioning.

H _5_: There is a significant positive relationship between the persistency of using scalable link building in SEO strategy with the success of online brand positioning.

## Methods

The research design is quantitative whereby a set of online survey questionnaires (See *Underlying data*) ^36^ was used to collect data randomly from online entrepreneurs who offer various products to customers. The respondents were contacted via Internet, websites, Facebook, and other social media sites and convenience sampling was used to selected 275 online entrepreneurs for this study. All the participant gave informed written consent to take part in this study.

The hypotheses were tested using Pearson Correlation analysis to determine causal relationships between variables, i.e., the positive or negative direction and strength of relationship based on the R-value, and at the significant of the relationship based on p-value.

## Results

The results shows that all the five hypotheses were accepted with the Pearson Correlation (R) values of more than 0.70 at significant values of less than 0.05.

**Figure 2.**
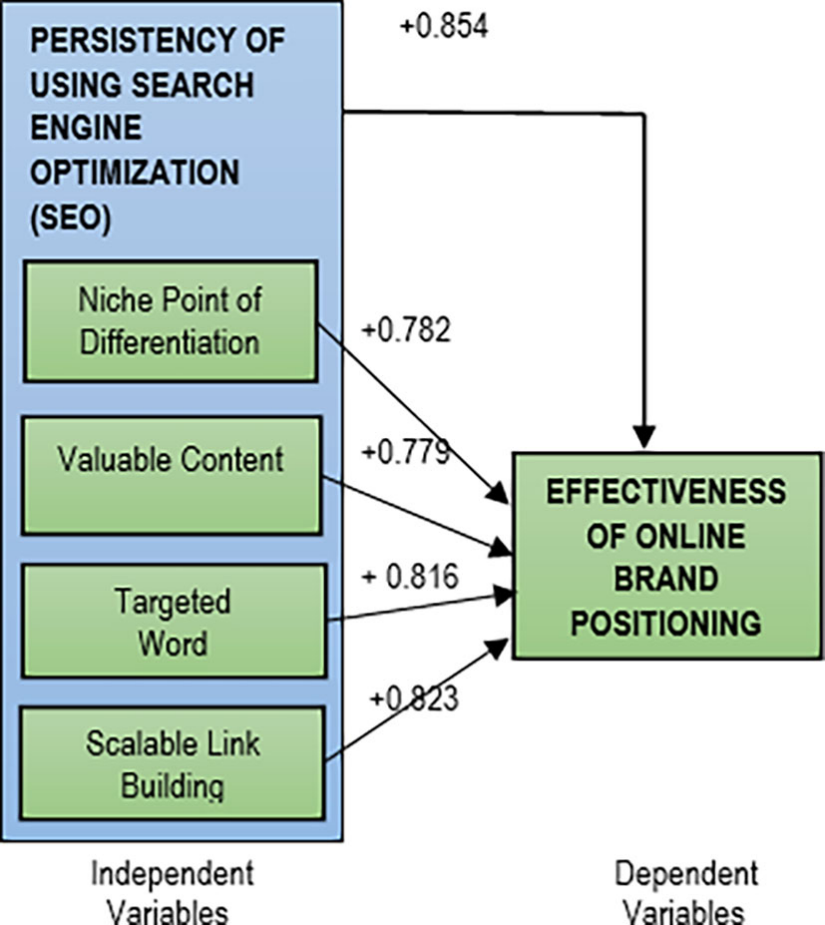
Pearson Correlation results.

All the relationships are positive and significantly strong. The Pearson Correlation results for relationships between persistency of using SEO strategy with the efficiency of online brand positioning are shown in Table 1 and Figure 2.

**Table 1.** Summary of Pearson Correlation results.

Variables	Pearson Correlation (R)	Direction of relationship	Strength
Search Engine Optimization (SEO)	+0.854	Positive	Strong (85.4%)
Niche point of differentiation	+0.782	Positive	Strong (78.2%)
Valuable content	+0.779	Positive	Strong (77.9%)
Targeted word	+0.816	Positive	Strong (81.6%)
Scalable link building	+0.823	Positive	Strong (83.3%)

Significant at p-value of 0.001.

## Discussions

The results show that the online entrepreneurs realise the effects of using the SEO strategy to enhance effectiveness of online brand positioning. Niche point of differentiation allows the online brand to appear distinctive and unique by making the brand appear more valuable and desirable to the customers. ^37^ Niche differentiation strategy gains competitive advantage by classifying the customers into smaller target, and later distinguish products for its excellent design, high awareness, easy accessibility, or other aspects. ^38^

Content can increase the SEO strategy components effectiveness, whereby niche consumers will be attracted to online brands with up-to-date content. ^39^ Content must have detailed brand information suitable for the target niche market, including a picture of the product/brand, ingredients, product usage and usefulness, warranty, returnable policies, companies’ details and other information; since without valuable content consumers may not trust the brands.

Integrating keywords can create a competitive advantage to online brand. ^40^ Good keywords provide direct natural traffics from the SEO strategy. ^41^ Keyword’s popularity can be determined by examining the search engine users’ behaviour. Focusing on how branded keywords across different markets, languages and search engines can also improve on international branding. ^41^^,^^42^

Scalable link building influences the effectiveness of online brand positioning by laddering technique. In searching for a brand information, consumers will start searching using general keywords that are not very popular, and later laddering the search to specific keyword. ^43^ Therefore in the SEO strategy, it is crucial for online entrepreneurs to ensure that the brand keywords appear on the first page of search engine and enhance the generic list of keywords to the specific ones gradually.

## Conclusions

This research has achieved its objectives in investigating the persistency of using the SEO strategy which includes niche point of differentiation, valuable content, targeted keyword, and scalable link building among online entrepreneurs. It contributes to new information on the online brand positioning, which can be utilized by online entrepreneurs. Nevertheless, the limitation of this study was that it only focused on the online entrepreneurs in Malaysia, therefore, future studies should investigate the real time usage of SEO platforms by the online entrepreneurs. Similarly, researchers can also investigate further appropriate training models to enhance online entrepreneurs’ knowledge and skills in using the SEO strategy as enablers in the branding market.

## Data availability

### Underlying data

Data Archiving and Networked Services (DANS): Search Engine Optimisation (SEO) strategy as determinants to enhance the online brand positioning. DOI: 10.17026/dans-xc5-jx74.

This project contains the following underlying data:

Data set information _SEO_Paper ID_ TIM21173_DIFCON2021 15102021.pdf. This is the data information sheet for this study QUESTIONNAIRE UMAR_SEO Paper ID TIM21173.pdf. This file contains the questionnaire used.

Data are available under the terms of the Creative Commons Zero “No rights reserved” Attribution 4.0 International (CC BY 4.0)

## Author contributions

U.F.A. worked on the idea and conceptualisation of the research topic, data curation and data analysis. J.M. was responsible on supervision of the research project, reviewing and editing work. N.A.B. worked on software and validation of findings.

## The ethical approval was obtained from the Research Ethics Committee of the DIFCON conference

Ethical Approval Number: EA1172021.
