# Supplementary material for: Search engine optimisation (SEO) strategy as determinants to enhance the online brand positioning
Source: F1000Res. 2024 Feb 1;11:714. Originally published 2022 Jun 28. [Version 2] doi: 10.12688/f1000research.73382.2 (PMC11066527; doi:10.12688/f1000research.73382.2)
Supplement: Peer review history

# Reviewer report: Reviewer response for version 2

**DOI:** 10.5256/f1000research.161641.r261167

**Published:** 2024-05-02

**Authors:** Anett Erdmann

**Affiliations:** 1 Department of Marketing, ESIC University, Madrid, Spain

The authors explore an interesting and relevant topic: how SEO improves brand positioning in the digital space. While the paper addresses relevant aspects for a persistent SEO strategy, there is room for improvement in the clarity of the study's delineation, methodological terms used, the presentation of the analytical results and the novelty for management.

- The paper's motivation should be backed up with numbers. Currently, it states that "many brand owners have opted to ignore the power of SEO in their online brand positioning strategy" and that "online business is relatively new," which, depending on the industry, may be outdated. SEO generates a large portion of a company's traffic share and has become even easier to manage with AI-based SEO optimization tools.
- The authors should clearly define what online brand positioning is and the importance of a long term perspective for online entrepreneurs. Positioning in the unbounded digital space can be understood in terms of Google's Search Engine Results Pages (SERP), any other search engine, positioning in any marketplace (e.g., Amazon), or a broad understanding of online positioning (incl. Social media) and brand awareness, but this delineation is not clear in the current version of the paper.
- Please also explain clearly the distinction between SEO and online advertising, which is mixed up in some parts of the current version of the paper.
- I recommend providing a table with the profile of the respondents. Given the sampling technique used, this would provide additional information for interpreting the results (in particular, which sectors are represented by the online entrepreneurs surveyed, if this information was available).
- Methodology: Using a Pearson Correlation analysis, you cannot speak of causality (revise your statement on p. 5), but only about the direction and strength/magnitude of the relationship. This should also be taken into account when interpreting the results. In addition to the sample estimates of the correlation, it is recommended to report the test statistics t=rxy/sqrt((1-rxy^2)/N-2) and the critical value of the t-distribution (here t(alpha=0.05, df=275-2)=1.96). Please also state the methodological approach in the introduction.
- Management implications for online entrepreneurs should be worked out in more detail, focusing on the persistence of SEO as it is the focus of your study. It may be useful to provide an executive summary with actionable insights.

Is the work clearly and accurately presented and does it cite the current literature?

Yes

If applicable, is the statistical analysis and its interpretation appropriate?

Partly

Are all the source data underlying the results available to ensure full reproducibility?

No

Is the study design appropriate and is the work technically sound?

Yes

Are the conclusions drawn adequately supported by the results?

Partly

Are sufficient details of methods and analysis provided to allow replication by others?

No

Reviewer Expertise:

Marketing analytics; Value assessment and pricing

I confirm that I have read this submission and believe that I have an appropriate level of expertise to confirm that it is of an acceptable scientific standard, however I have significant reservations, as outlined above.

# Reviewer report: Reviewer response for version 1

**DOI:** 10.5256/f1000research.77030.r148925

**Published:** 2022-10-04

**Authors:** Rizwan Raheem Ahmed

**Affiliations:** 1 Faculty of Management Sciences, Indus University, Karachi, Pakistan

The authors have tried their best to execute this paper, however, I have still the following reservations and suggestions for the sake of improvement of the undertaken study:

1. The logical sequence of the abstract should be 1) objective, 2) methodology, 3) findings, 4) conclusion, and 5) implication. Thus, the authors should rewrite the abstract in this manner.
2. The authors did not establish the motivation, significance, and novelty of the undertaken study. The authors are suggested to improve this important factor in the "Introduction" section. The introduction needs further details regarding the background, objectives, structure of the paper, etc. There is a lack of relevant and current citations in the introduction, the scientific paper needs appropriate, current, and relevant citations.
3. The literature should be presented in a separate section, it should be presented in an audit form, and it should be linked with the paper’s objectives, moreover, further relevant and current citations should be added to the introduction and previous literature.
4. The material and methods should be presented in a comprehensive manner. The conclusion should be elaborated in a comprehensive manner. The conclusion is always one step ahead of the findings. 
5. The practical, theoretical and societal implications should be discussed in a detailed manner after the conclusion, and in the light of the conclusion and results. 
6. The limitations and areas of future studies should be written at the end of the paper.
7. The spelling and grammatical mistakes should be corrected throughout the paper.

Is the work clearly and accurately presented and does it cite the current literature?

Yes

If applicable, is the statistical analysis and its interpretation appropriate?

Partly

Are all the source data underlying the results available to ensure full reproducibility?

Yes

Is the study design appropriate and is the work technically sound?

Yes

Are the conclusions drawn adequately supported by the results?

Yes

Are sufficient details of methods and analysis provided to allow replication by others?

No

Reviewer Expertise:

Management, marketing, advertising, digital marketing

I confirm that I have read this submission and believe that I have an appropriate level of expertise to confirm that it is of an acceptable scientific standard, however I have significant reservations, as outlined above.

# Response

**Published:** 2024-01-19

**Authors:** Junainah Mahdee

**Affiliations:** Multimedia University, Malaysia

The abstract sequence has been adjusted according to the F1000 format. We established the significance of research, added recent citations, elaborated the conclusion in a comprehensive manner with limitations/future research, and amended typo errors throughout the paper.

# Reviewer report: Reviewer response for version 1

**DOI:** 10.5256/f1000research.77030.r142588

**Published:** 2022-08-30

**Authors:** Umi Kartini Rashid

**Affiliations:** 1 Department of Technology and Management, Faculty of Technology Management and Business, Universiti Tun Hussein Onn Malaysia, Batu Pahat, Malaysia

For the discussion and conclusion part, H1 tested the four (4) major components of SEO as unidimensional, while H2~H5 tested them independently/multidimensional. So, what can you say about the differences between the results of unidimensional (combination of four components of SEO) and multidimensional? This paper would be more interesting if you could elaborate and justify further on these parts.

 I have included further comments by annotating the article, which you can find here.

Is the work clearly and accurately presented and does it cite the current literature?

Yes

If applicable, is the statistical analysis and its interpretation appropriate?

Yes

Are all the source data underlying the results available to ensure full reproducibility?

Yes

Is the study design appropriate and is the work technically sound?

Yes

Are the conclusions drawn adequately supported by the results?

Partly

Are sufficient details of methods and analysis provided to allow replication by others?

Yes

Reviewer Expertise:

Entrepreneurship, Business Management, Marketing, E-Commerce

I confirm that I have read this submission and believe that I have an appropriate level of expertise to confirm that it is of an acceptable scientific standard.
